# Patient Assessment and Therapy Planning Based on Homologous Recombination Repair Deficiency

**DOI:** 10.1016/j.gpb.2023.02.004

**Published:** 2023-02-14

**Authors:** Wenbin Li, Lin Gao, Xin Yi, Shuangfeng Shi, Jie Huang, Leming Shi, Xiaoyan Zhou, Lingying Wu, Jianming Ying

**Affiliations:** 1 Department of Pathology, National Cancer Center / National Clinical Research Center for Cancer / Cancer Hospital, Chinese Academy of Medical Sciences and Peking Union Medical College, Beijing 100021, China; 2 Geneplus–Shenzhen, Shenzhen 518000, China; 3 Institute of Microbiology, Chinese Academy of Sciences, Beijing 100101, China; 4 Geneplus–Beijing, Beijing 102206, China; 5 National Institutes for Food and Drug Control, Beijing 100050, China; 6 State Key Laboratory of Genetic Engineering, Human Phenome Institute, School of Life Sciences, Fudan University, Shanghai 200438, China; 7 Department of Pathology, Fudan University Shanghai Cancer Center, Shanghai 200032, China; 8 Department of Gynecologic Oncology, National Cancer Center / National Clinical Research Center for Cancer / Cancer Hospital, Chinese Academy of Medical Sciences and Peking Union Medical College, Beijing 100021, China

**Keywords:** DNA damage response, Homologous recombination repair deficiency, Poly(ADP-ribose) polymerase inhibitor, Biomarker, Harmonization

## Abstract

Defects in genes involved in the **DNA damage response** cause **homologous recombination repair deficiency** (HRD). HRD is found in a subgroup of cancer patients for several tumor types, and it has a clinical relevance to cancer prevention and therapies. Accumulating evidence has identified HRD as a **biomarker** for assessing the therapeutic response of tumor cells to **poly(ADP-ribose) polymerase inhibitors** and platinum-based chemotherapies. Nevertheless, the biology of HRD is complex, and its applications and the benefits of different HRD biomarker assays are controversial. This is primarily due to inconsistencies in HRD assessments and definitions (gene-level tests, genomic scars, mutational signatures, or a combination of these methods) and difficulties in assessing the contribution of each genomic event. Therefore, we aim to review the biological rationale and clinical evidence of HRD as a biomarker. This review provides a blueprint for the standardization and **harmonization** of HRD assessments.

## Introduction

Homologous recombination (HR) is an important repair mechanism for DNA double-strand breaks (DSBs). HR repair deficiency (HRD) is a cellular HR dysfunction that can be caused by germline/somatic mutations or epigenetic inactivation of HR-related genes. HRD has been found in many malignant tumors, especially in ovarian, breast, pancreatic ductal, and prostate cancers. HRD can be identified by genomic profiling as it induces specific and quantifiable genomic alterations. In addition, multiple studies have shown that HRD increases the sensitivity of tumors to poly(ADP-ribose) polymerase inhibitors (PARPIs). Therefore, HRD has become a biomarker for predicting the effects of PARPIs in patients with advanced ovarian cancer [1], [2], [3], [4]. Moreover, it can potentially be used as a biomarker to guide the clinical use of PARPIs and platinum-based chemotherapies in breast cancer [5], [6], prostate cancer [7], [8], and other cancer types [9], [10], [11]. This study reviews the definition of HRD; methodologies for HRD assessment; clinical applications, limitations, optimization, and standardization of HRD tests; and value of HRD tests as predictive and prognostic biomarkers in cancers. We aim to perform a comprehensive review to optimize and harmonize HRD assessment as an efficient biomarker for cancer detection and treatment.

## HRD definition

**Figure 1:**
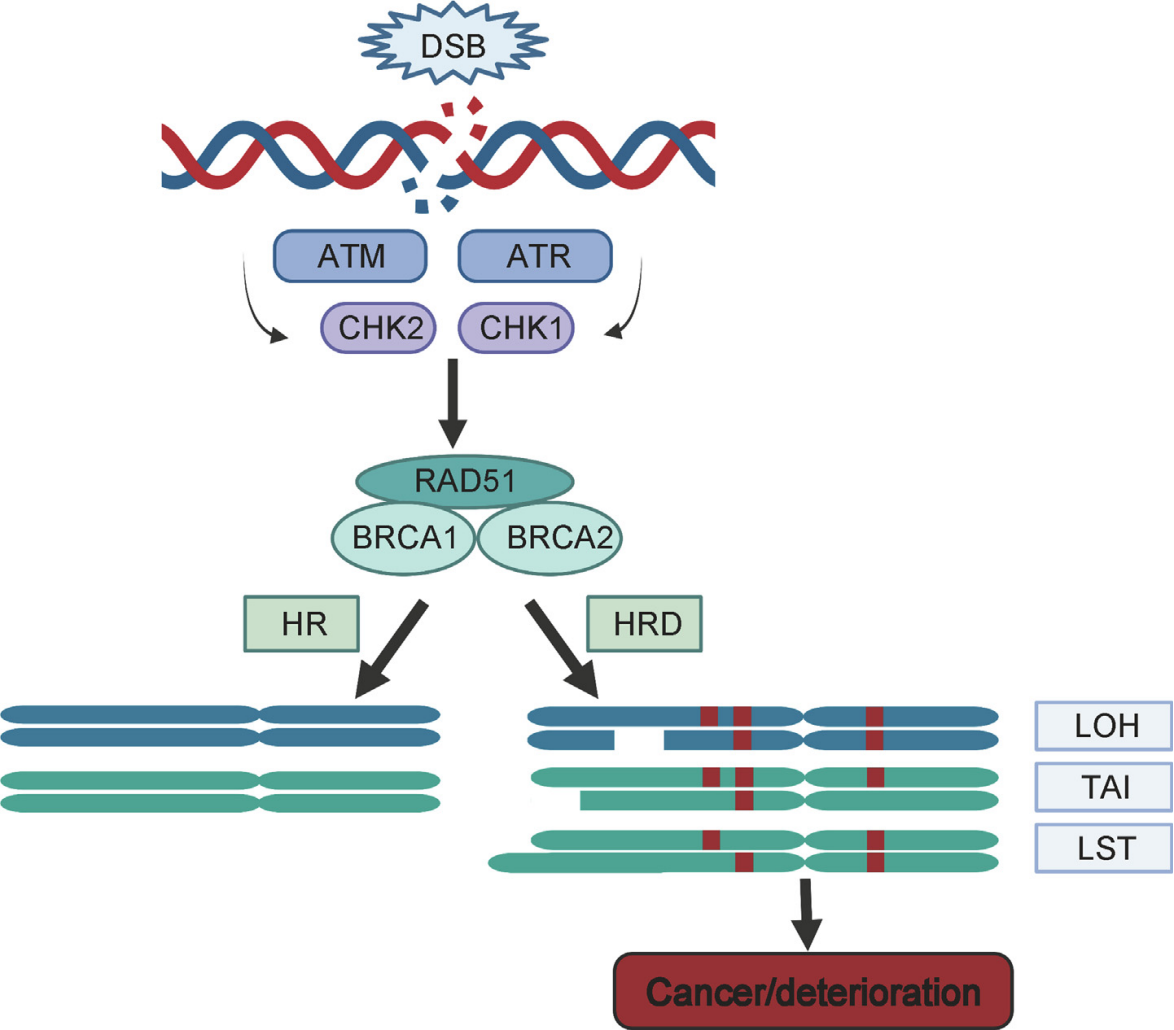
**HR and HRD occurring at DNA DSBs** HR is initiated after double-strand DNA breaks in a process that involves multiple proteins such as ATM, ATR, CHK1/2, RAD51, and BRCA1/2. HRD causes LOH, LST, TAI, and mutants (red boxes), which are manifestations of genomic instability that lead to cancer and deterioration. Created using BioRender.com. DSB, double-strand break; ATM, ataxia-telangiectasia mutated kinase; ATR, ataxia-telangiectasia-mutated-and-Rad3-related kinase; CHK1/2, checkpoint kinase 1/2; RAD51, radiation sensitive 51; BRCA1/2, breast cancer gene 1/2; HR, homologous recombination; HRD, homologous recombination repair deficiency; LOH, loss of heterozygosity; LST, large-scale state transition; TAI, telomeric allelic imbalance.

DNA damage can occur in many forms, including single-strand breaks and DSBs. The resulting high instability of the damaged DNA by DSBs can lead to gene mutations, cell apoptosis, and senescence. Therefore, DSB repair is crucial for maintaining DNA stability [12]. Various inter-connected pathways participate in DSB repair. HR repair can mend inter-strand crosslinks and DSBs using a complex, specific, and accurate mechanism [13], generating error-free DNA [3], [5]. Specifically, HR repair begins by recruiting the protein kinase ataxia-telangiectasia mutated kinase (ATM), through the meiotic recombination 11 (MRE11)–radiation sensitive 50 (RAD50)–Nijmegen breakage syndrome 1 (NBS1) complex at DSB sites. ATM then phosphorylates downstream proteins such as breast cancer gene 1 (BRCA1) and cyclin-dependent kinases (CDKs), promoting BRCA1 activation and initiating DSB repair. Hence, BRCA2, partner and localizer of BRCA2 (PALB2), replication protein A (RPA), and RAD51 induce HR repair. In the repair process of the damaged area of the DNA strand, the homologous region of the sister chromatid is used as a template [12]. Most HRD-induced DSB repairs use microhomology-mediated end-joining, non-homologous end-joining, or single-strand annealing [14]. However, these mechanisms have low fidelity and are prone to errors during DNA repair. Misrepaired or unrepaired DSBs can promote the accumulation of genomic alterations, including copy number variants, insertions, deletions, or structural rearrangements of chromosomes. Such alterations can cause genomic instability and lead to cancer and deterioration of tumors. These features of genomic instability are known as genomic scars [15] (Figure 1). HR-related gene mutations are prevalent in various cancers. For instance, deleterious somatic/germline alterations in *BRCA1/2* (essential components of the HR pathway) are the most common indicators of HRD. Importantly, HRD is also prevalent in tumors that harbor non-*BRCA* mutations in the HR pathway, thus generating a *BRCA*-like phenotype [16]. Multiple studies have shown that HRD increases the sensitivity of tumors to various tumor-targeting drugs such as PARPIs, platinum-based chemotherapies, mitomycin C, and alkylating agents [17], [18], [19], [20], [21]. There is evidence of HRD in many malignant tumors, especially in ovarian [22], [23], breast [17], [24], [25], pancreatic ductal [26], [27], [28], and prostate cancers [29]. The HRD status of various cancer types [30], [31] is summarized in Table 1.

**Table 1:** **HRD in tumors**

**HRD status**	**Tumor**	**HRD percentage in patients**	**Refs.**
Somatic/germline mutations in *BRCA1/2*; other HR-related mutations	Ovarian cancer	13%–15% with germline *BRCA1/2* mutations 20% with somatic *BRCA1/2* mutations 30% with HR-related mutations 50% in HGSOC patients with HRD	[22], [23]
	Breast cancer	5%–10% with germline *BRCA1/2* mutations > 20% with HRD 10%–20% in TNBC patients with germline *BRCA1/2* mutations 3%–5% in TNBC patients with somatic *BRCA1/2* mutations	[17], [24], [25]
	Pancreatic cancer	5%–9% with HRD	[26], [27], [28]
	Prostate cancer	9.9% with HRD	[31]
Somatic/germline mutations in *BRCA1/2*; other HR-related mutations; *BRCA1* methylation	Tenosynovial giant cell tumor	12.5% with HRD	[30]
	Bladder urothelial carcinoma	7.1% with HRD	[30]
	Stomach and esophageal carcinoma	5.3% with HRD	[30]
	Lung squamous cell carcinoma	4.5% with HRD	[30]
	Sarcoma	4.2% with HRD	[30]
	Skin cutaneous melanoma	3.7% with HRD	[30]
	Cervical squamous cell carcinoma and endocervical adenocarcinoma	3.6% with HRD	[30]
	Adrenocortical carcinoma	3.3% with HRD	[30]
	Uterine corpus endometrial carcinoma	3.1% with HRD	[30]
	Lung adenocarcinoma	2.7% with HRD	[30]
	Colorectal cancer	2.3% with HRD	[30]
	Head and neck squamous cell carcinoma	1.8% with HRD	[30]
	Liver hepatocellular carcinoma	1.8% with HRD	[30]

*Note*: BRCA, breast cancer gene; HR, homologous recombination; HRD, HR repair deficiency; HGSOC, high-grade serous ovarian cancer; TNBC, triple-negative breast cancer.

## Methodologies for HRD assessment

Over the past decade, various studies have focused on the genome fingerprints caused by HRD in tumors. Studies have also focused on identifying the factors that predict the response of tumors to HRD-based therapies [32].

The HRD score is a score that quantifies the genomic instability status of tumors caused by an abnormal HR pathway. HRD induces specific and quantifiable genomic alterations, including mutations, chromosomal structural abnormalities, and copy number variants, which are the theoretical basis for current HRD clinical tests [33], [34]. However, there are no unified standards for HRD testing. The two main categories of methods currently used for assessing the genomic instability status of a patient are genomic scar analysis based on single-nucleotide polymorphisms (SNPs) and single- or multi-dimensional genomic profiles obtained from whole-genome sequencing (WGS) data, which are described below.

The first category uses genomic scar analysis based on SNPs. In this category, HRD scores are mainly computed from the results of high-density SNP arrays of the whole genome or genomic SNP backbone probes based on next-generation sequencing (NGS). These HRD scores include the degree of genomic instability and mutations in HR-associated genes [25]. Briefly, HR-related mutations, such as *BRCA1/2* deleterious mutations and promoter methylation, are detected in tumor cells. The genomic instability caused by these molecular mechanisms is analyzed using high-density SNP loci, which detect copy number variation indicators such as loss of heterozygosity (LOH; found in genomic regions containing only one of the two parental copies), large-scale state transition (LST; genomic breaks between adjacent genomic regions > 10 Mb), and telomeric allelic imbalance (TAI; allelic imbalance extending to the subtelomere but not crossing the centromere). This information is then integrated to calculate HRD scores [35], [36], [37], [38], [39]. The threshold of the HRD scores is then defined according to the efficacy of PARPI- and/or platinum-based chemotherapies [40].

**Figure 2:**
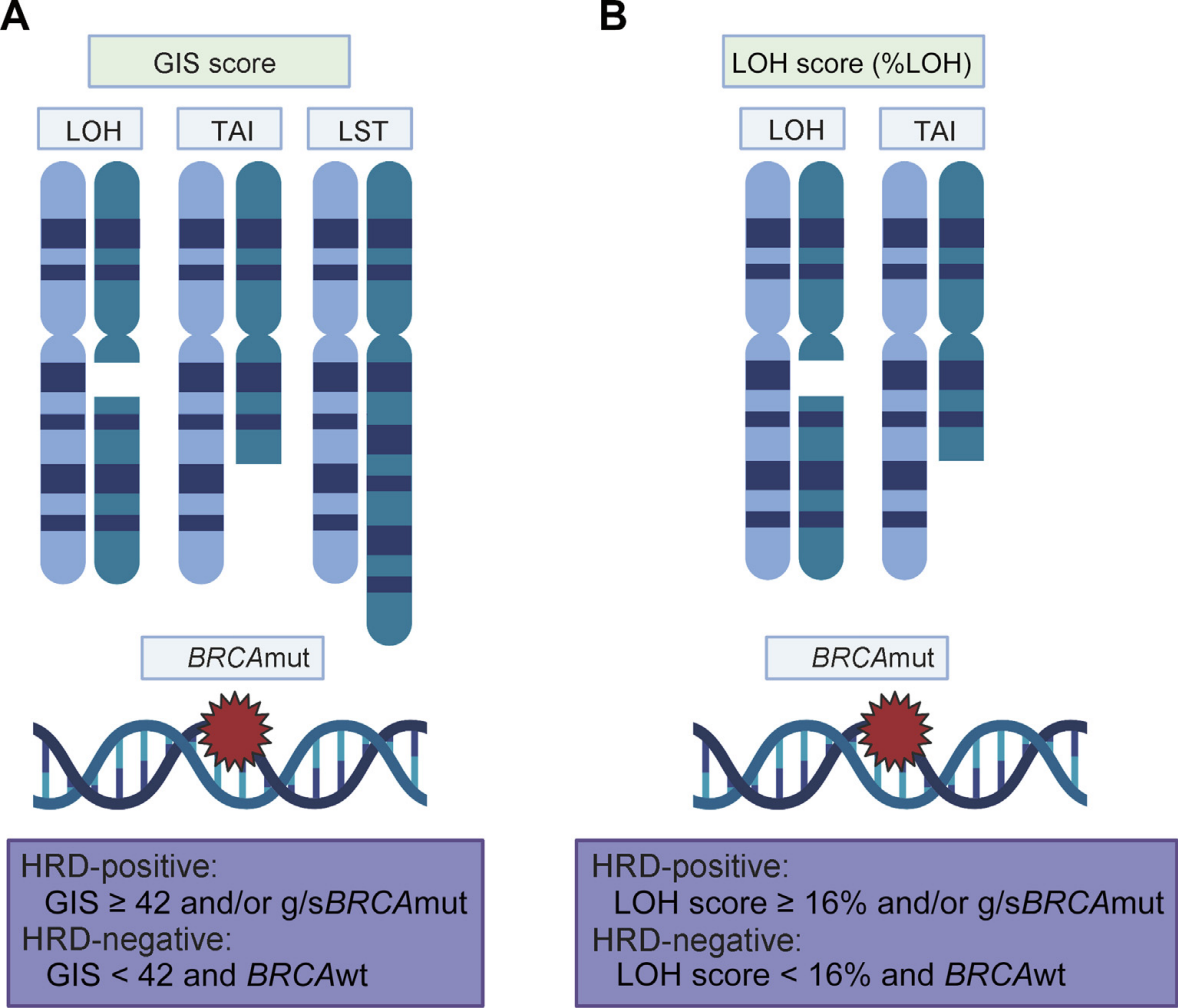
**HRD assays based on genomic scars** **A.** myChoice CDx can quantify HRD levels using GIS, which combines LOH, TAI, and LST, using DNA isolated from FFPE tumor tissue specimens. **B.** FoundationFocus CDx*_BRCA_*_LOH_ evaluates HRD genomic scars by assessing the percentage of segments with LOH in the whole genome covering selected SNPs on 22 chromosomes. Created using BioRender.com. GIS, genomic instability score; FFPE, formalin-fixed paraffin-embedded; SNP, single-nucleotide polymorphism; g/s*BRCA*mut, germline/somatic *BRCA* mutation; *BRCA*wt, *BRCA* wild-type; %LOH, percentage of LOH.

Currently, the only two commercial tests approved by the United States Food and Drug Administration (FDA) for assessing the status of HRD based on SNPs are the myChoice CDx (Myriad Genetics) and FoundationFocus CDx*_BRCA_*_LOH_ (Foundation Medicine) assays. These two assays use different assessment methods. myChoice CDx identifies HRD status using NGS. It analyzes the coding regions and population-specific SNPs of *BRCA1/2*. The assessment of the HRD scores of myChoice CDx is based on the degree of somatic copy number variants (SCNVs) in tumors. Therefore, the accuracy of HRD scores is strongly associated with the accuracy of SCNVs in tumors. myChoice CDx quantifies HRD levels using the genomic instability score (GIS), which is based on a combination of the copy number variation indicators LOH, TAI, and LST derived from DNA isolated from formalin-fixed paraffin-embedded (FFPE) tumor tissue specimens [41]. In addition, tumor cell purity and whole tumor ploidy [42] are essential factors for improving the detection accuracy of segment copy numbers. The typical threshold score of myChoice HRD is 42 [41]. Specifically, tumors are considered HRD-positive if they had a high myChoice HRD score (GIS ≥ 42) and/or pathogenic *BRCA1/2* mutations, whereas HRD-negative tumors are those with a low myChoice HRD score (GIS < 42) and wild-type *BRCA1/2*. The threshold choice is based on the HRD scores of the fifth percentile of biallelic-inactivated *BRCA* ovarian and breast cancer samples, and it was shown to be effective in predicting the sensitivity to platinum-based chemotherapies in breast cancer [41], [43] (Figure 2A). The other assay, FoundationFocus CDx*_BRCA_*_LOH_, evaluates HRD genomic scars by assessing the percentage of segments with LOH in the whole-genome covering selected SNPs on 22 chromosomes. The threshold used by the FoundationFocus CDx*_BRCA_*_LOH_ assay is 16%, which was set after several adjustments based on the results of clinical trials, such as REAL3 for platinum and ARIEL2 and ARIEL3 for recapture. According to the FoundationFocus CDx*_BRCA_*_LOH_ assay, an HRD-positive patient has a tumor *BRCA1/2* mutation and/or a genomic LOH score ≥ 16%, whereas an HRD-negative patient has a wild-type *BRCA1/2* and a genomic LOH score < 16% [44], [45] (Figure 2B).

The other main category of methods currently used for assessing the genomic instability status of a patient is based on single- or multi-dimensional genomic profiles obtained from WGS data. This category includes the detection of specific mutational signatures such as Signature 3 and HRD detection models such as HRDetect. Signature 3 is identified as a mutational signature of many deletions at breakpoint sites overlapping with microhomology. In various types of cancer, Signature 3 is related to mutations in *BRCA1/2* and methylation of the *BRCA1* promoter [46], [47], and it has been also associated with tumor sensitivity to platinum [48]. Nevertheless, the usage of Signature 3 as a marker of HRD has some limitations, such as low diagnostic specificity and lack of exact thresholds. Moreover, it is not an adequate biomarker for predicting PARPI efficacy in tumor cells [49]. Given these limitations of HRD detection based on a single biomarker, WGS-based assays that optimize HRD assessment by incorporating more types of genes and chromosomal structural alterations have been developed. These include HRDetect [50], a classifier containing six mutational signatures based on WGS, which was designed to predict *BRCA1/2* deficiency. The six mutational signatures are microhomology-mediated indels, the HRD index, base substitution signature 3 (enriched in C > G substitutions), rearrangement signature 3 (short < 10-kb tandem duplications), rearrangement signature 5 (non-clustered deletions < 100 kb), and base substitution signature 8 (enriched in C > A substitutions). These signatures were assigned specific weights using a machine learning algorithm and then integrated into a single HRDetect score [12], [50], [51]. Additionally, HRDetect can identify mutational phenotypes similar to *BRCA1/2* deficiency in non-*BRCA* mutation tumors, a phenomenon known as *BRCA*ness [41]. HRDetect can assess the efficacy of PARPI in patients with *BRCA1/2* defects [50]. The cut-off value of HRDetect should be computed based on the sensitivity of cells to platinum- and PARPI-based therapies, and it could be used to predict the responses to such therapies of patients. However, insufficient clinical results support HRDetect as a biomarker to predict PARPI efficacy. The methods of HRD assessment described above are summarized in Table 2.

**Table 2:** **Methods of HRD assessment**

**Method for HRD assessment**		**Diagnostic technique**	**Definition**	**Refs.**
Genomic scar	myChoice CDx (Myriad Genetics)	High-density SNP arrays based on NGS	GIS combined with LOH, TAI, and LST; pathogenic *BRCA1/2* mutations	[41], [43]
	FoundationFocus CDx*_BRCA_*_LOH_ (Foundation Medicine)	High-density SNP arrays based on NGS	Percentage of segments with LOH in the whole genome; pathogenic *BRCA1/2* mutations	[44], [45]
Mutational signature	Signature 3	WGS	A large number of deletions at the breakpoint sites overlapping with microhomology	[46], [47]
	HRDetect	WGS	A classifier with six mutational signatures, including microhomology-mediated indels, HRD index, base substitution signature 3, rearrangement signature 3, rearrangement signature 5, and base substitution signature 8	[12], [50], [51]

*Note*: NGS, next-generation sequencing; WGS, whole-genome sequencing; GIS, genomic instability score; LOH, loss of heterozygosity; LST, large-scale state transition; TAI, telomeric allelic imbalance; SNP, single-nucleotide polymorphism.

## Clinical applications of HRD testing

HRD leads to defective DNA DSB damage repair. This makes cells with HRD highly sensitive to DNA-break-inducing platinum drugs and PARPIs, which can lead to synthetic lethality. Specifically, platinum drugs enter the nucleus and form Pt–DNA compounds, which cause structural changes in DNA and impair DNA replication and transcription, resulting in apoptosis. In addition, PARPIs can halt the DNA repair process that is governed by PARP1, which is involved in DNA damage repair by excising bases in single-strand DNA breaks. The halt in the repair of damaged DNA can, thus, lead to severe genomic instability, causing lethality in tumors with pathogenic *BRCA1/2* mutations or other HRD-associated mutations [33], [52], [53]. HRD has extensive applications as a biomarker of tumor responsivity to therapeutic agents that target DNA damage and has achieved good results in clinical trials for ovarian cancer [1]. It may also guide the clinical use of PARPI- and platinum-based drugs for breast, pancreatic, prostate, and other cancers [10], [25], [43], [54], [55]. Clinical-grade HRD analysis, through assays such as FoundationFocus CDx*_BRCA_*_LOH_ and myChoice CDx, has been approved as a companion diagnostic test for HRD-positive patients. For example, HRD is considered a frequently used biomarker for ovarian cancer that has guided the development of specific treatments, such as PARPIs, showing better outcomes for patients with high levels of HRD [53].

Multiple clinical trials have evaluated HRD as a prognostic biomarker for ovarian cancer. For instance, the SOLO2 trial showed that olaparib could significantly improve the median progression-free survival (PFS) of patients with epithelial ovarian cancer (EOC) with germline *BRCA* mutations [1]. The Study 19 trial corroborated the conclusion of the SOLO2 trial and showed that PFS was much longer in the olaparib group with *BRCA* mutations than that in the group with wild-type *BRCA* [56]. Furthermore, the PAOLA-1 trial demonstrated that the conclusion of Study 19 also applies to HRD-positive patients with EOC [2]. The ARIEL3 trial showed that after rucaparib treatment, HRD-positive patients with EOC had a significantly longer median PFS than did HRD-negative patients [45]. NOVA [3] and PRIMA [57] clinical trials have shown that niraparib can significantly improve the median PFS of HRD-positive patients with EOC compared with HRD-negative patients. Furthermore, the VELIA trial demonstrated that adding a veliparib therapy significantly prolonged the median PFS of patients with HRD-positive EOC [4]. Finally, the SCOTROC4 trial showed that platinum-based therapy improved the median PFS of patients with HRD-positive ovarian cancer. [58]. These clinical trials have demonstrated that HRD is an effective biomarker for the response of ovarian cancer to treatment. They also showed that PARPIs could significantly improve the PFS of HRD-positive patients with ovarian cancer.

Several studies have also been conducted on HRD in breast cancer. The OlympiAD trial showed that the median PFS was significantly longer in the olaparib group than that in the control group (treated with capecitabine, eribulin, or vinorelbine) of patients with *HER2*-negative metastatic breast cancer and germline *BRCA* mutations, and the response rate was much higher in the olaparib group than that in the control group [5]. A clinical trial analysis including Cisplatin-1, Cisplatin-2, and PrECOG 0105 showed that with platinum-containing therapies, HRD-positive patients with triple-negative breast cancer (TNBC) had significantly better residual cancer burden scores (RCB) and pathological complete response (pCR) [41]. GeparSixto also showed that HRD-positive TNBC patients had better pCR during carboplatin-added neoadjuvant therapies and that carboplatin significantly prolonged the disease-free survival (DFS) of HRD-positive patients [59]. Telli et al. showed that HRD-positive patients with TNBC responded significantly better to standard neoadjuvant chemotherapies, including DNA-damaging agents such as anthracycline, than did HRD-negative patients [60]. In the BrighTNess trial, TPV-AC (paclitaxel, carboplatin, veliparib, doxorubicin, and cyclophosphamide) yielded better results in the HRD-positive group (HRD ≥ 33 or 42; pCR: 60.8% or 61.7%) than in the HRD-negative group (HRD < 33 or 42; pCR: 33.3% or 36.1%) [6]. These clinical trials have shown that HRD is a promising biomarker for the response to treatment of breast cancer.

A few studies have shown that 5%–9% of patients with pancreatic cancer are HRD-positive [26], [27], [28]. However, most HRD-related drugs are not yet used as routine therapies against pancreatic cancer. Several studies have investigated the effect of HRD status on prognosis and the efficacy of platinum therapies in pancreatic cancer. For example, a study showed that patients with pancreatic cancer with HRD were more sensitive to platinum-based chemotherapies and have better prognoses than patients without HRD [9]. O’Reilly et al. showed that therapies based on cisplatin and veliparib could dramatically improve the median overall survival (OS) and the overall response rate (ORR) of HRD-positive patients with advanced pancreatic ductal adenocarcinoma [10]. The POLO trial showed that the median PFS was significantly longer in the olaparib group than in the placebo group of patients with metastatic pancreatic cancer and germline *BRCA1/2* mutations [11]. Finally, a meta-analysis and systematic review showed that HRD might improve the effect of platinum-based chemotherapies and prolong the median OS in patients with resected and metastatic pancreatic cancer treated with platinum-based chemotherapies. However, more clinical trials are needed to verify these conclusions [18].

HRD is a promising biomarker for guiding PARPI treatment in patients with metastatic castration-resistant prostate cancer (mCRPC). In the clinical trial NCT01972217, the median radiographic PFS in the olaparib group (17.8 months) was much longer than that in the placebo group (6.5 months) in patients with HR mutations [55]. The GALAHAD trial demonstrated that niraparib treatment in patients with mCRPC resulted in significantly better ORR and complete response rate (CRR) in HRD-positive patients [61]. In the PROfound trial, the median OS and imaging-based median PFS were significantly longer in the olaparib group than that in the control group (treated with enzalutamide or abiraterone) of patients with mCRPC with at least one mutation in *BRCA1*, *BRCA2*, or *ATM* [8], [62]. The key HRD-associated clinical trials are summarized in Table 3.

**Table 3:** **Key clinical trialsinvolv**ing**HRD**

**Study**	**Cancer type**	**Treatment strategy**	**Biomarker**	**Subgroup**	**Main result**	**Refs.**
SOLO2 (NCT01874353)	EOC	Olaparib *vs.* placebo	BRACAnalysis test (Myriad Genetics): g*BRCA*mut	HRD^+^ (g*BRCA*mut)	HR: 0.30 Median PFS: 19.1 *vs.* 5.5 (*P* < 0.0001)	[1]
Study19 (NCT00753545)	EOC	Olaparib *vs.* placebo	Foundation Medicine: t*BRCA*mut	HRD^+^ (*BRCA*mut)	HR: 0.18 Median PFS: 11.2 *vs.* 4.3 (*P* < 0.0001)	[56]
				HRD^−^ (*BRCA*wt)	HR: 0.54 Median PFS: 7.4 *vs.* 5.5 (*P* = 0.0075)	
ARIEL3 (NCT01968213)	EOC	Rucaparib *vs.* placebo	Foundation Medicine T5 NGS assay and BRACAnalysis CDx test (Myriad Genetics): (1) g/s*BRCA*mut (2) LOH-high (LOH score ≥ 16%) LOH-low (LOH score < 16%)	HRD^+^ (g/s*BRCA*mut)	HR: 0.23 Median PFS: 16.6 *vs.* 5.4 (*P* < 0.0001)	[45]
				HRD^+^ (g/s*BRCA*mut or LOH-high)	HR: 0.32 Median PFS: 13.6 *vs.* 5.4 (*P* < 0.0001)	
				HRD^−^ (LOH-low and *BRCA*wt)	HR: 0.58 Median PFS: 6.7 *vs.* 5.4 (*P* = 0.0049)	
NOVA (NCT01847274)	EOC	Niraparib *vs.* placebo	BRACAnalysis test (Myriad Genetics): g*BRCA*mut myChoice HRD (Myriad Genetics): GIS-high (GIS ≥ 42) GIS-low (GIS < 42)	HRD^+^ (g*BRCA*mut)	HR: 0.27 Median PFS: 21 *vs.* 5.5 (*P* < 0.001)	[3]
				HRD^+^ (GIS-high and g*BRCA*wt)	HR: 0.38 Median PFS: 12.9 *vs.* 3.8 (*P* < 0.001)	
				HRD^−^ (g*BRCA*wt)	HR: 0.45 Median PFS: 9.3 *vs.* 3.9 (*P* < 0.001)	
				HRD^−^ (GIS-low and g*BRCA*wt)	HR: 0.58 Median PFS: 6.9 *vs.* 3.8 (*P* = 0.02)	
PRIMA (NCT02655016)	EOC	Niraparib *vs.* placebo	myChoice test (Myriad Genetics): (1) GIS-high (GIS ≥ 42) GIS-low (GIS < 42) (2) t*BRCA*mut	HRD^+^ (t*BRCA*mut)	HR: 0.4 Median PFS: 22.1 *vs.* 10.9 (*P* < 0.001)	[57]
				HRD^+^ (GIS-high or t*BRCA*mut)	HR: 0.43 Median PFS: 21.9 *vs.* 10.4 (*P* < 0.001)	
				HRD^+^ (GIS-high and t*BRCA*wt)	HR: 0.5 Median PFS: 19.6 *vs.* 8.2 (*P* = 0.006)	
				HRD^−^ (GIS-low and t*BRCA*wt)	HR: 0.68 Median PFS: 8.1 *vs.* 5.4 (*P* = 0.02)	
PAOLA-1 (NCT02477644)	EOC	Olaparib + bevacizumab *vs.* placebo + bevacizumab	myChoice HRD Plus assay (Myriad Genetics): (1) GIS-high (GIS ≥ 42) GIS-low (GIS < 42) (2) t*BRCA*mut	HRD^+^ (t*BRCA*mut)	HR: 0.31 Median PFS: 37.2 *vs.* 21.7	[2]
				HRD^+^ (GIS-high or t*BRCA*mut)	HR: 0.33 Median PFS: 37.2 *vs.* 17.7	
				HRD^+^ (GIS-high and t*BRCA*wt)	HR: 0.43 Median PFS: 28.1 *vs.* 16.6	
				HRD^−^ (t*BRCA*wt)	HR: 0.71 Median PFS: 18.9 *vs.* 16	
				HRD^−^ (GIS-low) or unknown	HR: 0.92 Median PFS: 16.9 *vs.* 16	
VELIA (NCT0247058)	EOC	Carboplatin/taxane + maintenance placebo *vs.* carboplatin/taxane + veliparib + maintenance veliparib	BRACAnalysis CDx or myChoice HRD CDx assay (Myriad Genetics): (1) GIS-high (GIS ≥ 33) GIS-low (GIS < 33) (2) t*BRCA*mut	HRD^+^ (t*BRCA*mut)	HR: 0.44 Median PFS: 37.4 *vs.* 22 (*P* < 0.001)	[4]
				HRD^+^ (GIS-high or t*BRCA*mut)	HR: 0.57 Median PFS: 31.9 *vs.* 20.5 (*P* < 0.001)	
				HRD^−^ (t*BRCA*wt)	HR: 0.8 Median PFS: 18.2 *vs.* 15.1	
				HRD^−^ (t*BRCA*wt and GIS-low)	HR: 0.81 Median PFS: 15.0 *vs.* 11.5	
SCOTROC4 (NCT00098878)	EOC	Carboplatin	Genome-wide SNP data; sum of LOH, TAI, and LTS score: (1) HRD score ≥ 42 or HRD score ≥ 33 (2) t*BRCA*mut	HRD^+^ (t*BRCA*mut or HRD score ≥ 42/ HRD score ≥ 33) *vs.* HRD^−^ (t*BRCA*wt and HRD score < 42/HRD score < 33)	HR: 0.50 Median PFS: 16.5 *vs.* 9.5 (*P* < 0.001) (HRD ≥ 42) HR: 0.51 (HRD ≥ 33)	[58]
				HRD^+^ (t*BRCA*mut) *vs.* HRD^−^ (t*BRCA*wt)	HR: 0.48 Median PFS: 18.9 *vs.* 11.6 (*P* = 0.0017)	
OlympiAD (NCT02000622)	HER2^−^ metastatic BC	Olaparib *vs.* chemotherapy (capecitabine, vinorelbine, eribulin)	BRACAnalysis (Myriad Genetics): g*BRCA*mut	HRD (g*BRCA*mut)	Median PFS: 7.0 *vs.* 4.2 Response rate: 59.9% *vs.* 28.8%	[5]
Cisplatin-1 (NCT00148694), Cisplatin-2 (NCT00580333), PrECOG 0105 (NCT00813956)	TNBC	Carboplatin + gemcitabine + iniparib	Genome-wide SNP data; sum of LOH, TAI, and LTS score: (1) HRD-high ≥ 42 HRD-low < 42 (2) t*BRCA*mut	HRD^+^ (HRD-high and/or t*BRCA*mut) *vs.* HRD^−^ (HRD-low and t*BRCA*wt)	RCB0/1: 68% *vs.* 30% OR: 4.96 (*P* < 0.01) pCR: 42% *vs.* 10% OR: 6.52 (*P* < 0.01)	[41]
		Cisplatin + bevacizumab			RCB0/1: 51.7% *vs.* 9.5% OR: 10.18 (*P* < 0.01) pCR: 27.5% *vs.* 0% OR: 17 (*P* < 0.01)	
GeparSixto (NCT01426880)	TNBC	Paclitaxel + doxorubicin + bevacicumab *vs.* Paclitaxel + doxorubicin + bevacicumab + carboplatin	Myriad Genetics: (1) HRD-high ≥ 42 HRD-low < 42 (2) t*BRCA*mut	HRD^+^ (HRD-high or t*BRCA*mut)	pCR: 33.9% *vs.* 63.5% OR: 3.4 (*P* < 0.01)	[59]
				HRD^+^ (HRD-high)	pCR: 31.7% *vs.* 63.2% OR: 3.69 (*P* < 0.01)	
				HRD^+^ (HRD-low and t*BRCA*wt/uncertain)	pCR: 20.0% *vs.* 29.6% OR: 1.7	
Telli et al.	TNBC and ER^+^ and/or PR^+^/HER2^−^ BC with *BRCA1/2*mut	Anthracycline, taxane, or anthracycline + taxane-based neoadjuvant chemotherapy	Genome-wide SNP data; sum of LOH, TAI, and LTS score: (1) HRD-high ≥ 42 HRD-low < 42 (2) t*BRCA*mut	HRD^+^ (HRD-high or t*BRCA*mut)	RCB0/1: 63% OR: 5.1 (*P* < 0.01) pCR: 41% OR: 13.06 (*P* < 0.01)	[60]
				HRD^+^ (HRD-high)	RCB0/1: 77% OR: 10 (*P* < 0.01) pCR: 46% OR: 16.29 (*P* < 0.01)	
				HRD^−^ (HRD-low and t*BRCA*wt)	RCB0/1: 25% pCR: 5%	
O’Reilly et al.	Advanced PDAC	Gemcitabine + cisplatin + veliparib	Myriad Genetics: g*BRCA*mut	HRD^+^ (g*BRCA*mut) *vs.* HRD^−^ (*BRCA*wt)	Median OS: 23.3 *vs.* 11 ORR: 77.8% *vs.* 0%	[10]
POLO (NCT02184195)	mPC	Olaparib tablets *vs.* placebo	BRACAnalysis CDx test (Myriad Genetics): g*BRCA*mut	HRD^+^ (g*BRCA*mut)	HR: 0.53 Median PFS: 7.4 *vs.* 3.8 (*P* < 0.01)	[11]
GALAHAD (NCT02854436)	mCRPC	Niraparib		HRD^+^ (biallelic *BRCA* mutation) *vs.* HRD^−^ (*BRCA*wt)	ORR: 41% *vs.* 9% CRR: 63% *vs.* 17%	[61]
PROfound (NCT02987543)	mCRPC	Olaparib *vs.* enzalutamide or abiraterone	FoundationOne CDx: *BRCA*mut or *ATM* mut	HRD^+^ (*BRCA*mut or *ATM*mut)	HR: 0.69 Median OS: 19.1 *vs.* 14.7 (*P* = 0.02) HR: 0.34 Imaging-based median PFS: 7.4 *vs.* 3.6 (*P* < 0.01)	[8], [62]

*Note*: g/s*BRCA*mut, germline/somatic *BRCA* mutation; g*BRCA*mut, germline *BRCA* mutation; t*BRCA*mut, tumor *BRCA* mutation; g*BRCA*wt, germline *BRCA* wild-type; t*BRCA*wt, tumor *BRCA* wild-type; *BRCA*mut, *BRCA* mutation; *BRCA*wt, *BRCA* wild-type; *ATM*mut, *ATM*mutation; PFS, progression-free survival; OS, overall survival; ORR, objective response rate; CRR, complete response rate; RCB, residual cancer burden; pCR, pathologic complete response; OR, odd ratio; HR, hazard ratio; HRD^+^, HRD-positive; HRD^−^, HRD-negative; GIS, genomic instability score; ATM, ataxia-telangiectasia mutated kinase; EOC, epithelial ovarian cancer; BC, breast cancer; PDAC, pancreatic ductal adenocarcinoma; mPC, metastatic prostate cancer; mCRPC, metastatic castration-resistant prostate cancer.

HRD is still in its early stages of development as a pan-cancer biomarker in clinical applications. In particular, the correlation between HRD and immune checkpoint inhibitors in pan-cancer warrants further investigation. HRD tumors have been considered more immunogenic owing to their increased tumor mutation burden (TMB) and type I Interferon (IFN) genes. Therefore, HRD tumors may be more susceptible to checkpoint inhibitor therapies [63]. In breast cancer, low-level expression of *BRCA1*, *ATM*, and *XRCC1* mutations significantly correlated with higher CD8^+^ T-cell infiltration [64], [65]. Moreover, *BRCA* deficiency was related to elevated PD-L1 expression [66], [67] and T-cell infiltration in ovarian cancers [66]. In the MEDIOLA trial, the combination of olaparib and durvalumab (a PD-L1 inhibitor) showed a good ORR (68%) in patients with ovarian cancer and germline *BRCA* mutations [68]. Therefore, the use of immune checkpoint inhibitors may increase the benefit of HRD-positive patients with cancer from platinum- and PARPI-based treatments. Multiple clinical trials based on combination therapies of platinum, PARPIs, and immune checkpoint inhibitors are ongoing [69].

## Challenges of HRD testing

Currently, there is no unified gold standard for assessing HRD. HRD is commonly assessed by evaluating the genomic features of tumors harboring deleterious HR-related mutations, such as *BRCA1/2* or genomic scars, which can indicate genomic instability. However, clinical trials have identified patients with HRD-negative or no deleterious HR mutations that respond well to PARPI treatment [45], [70], [71], suggesting that more accurate assessments methods of HRD are required. Currently, the challenges in the clinical application of HRD are mainly in the following four areas.

First, there is a need to verify whether non-*BRCA* mutations or promoter methylation of the HR pathway can be used as biomarkers for guiding tumor treatment. Only germline or somatic *BRCA* mutations have been shown to successfully predict the efficacy of PARPIs treatment in clinical practice. In contrast, there are insufficient clinical trial results to prove that non-*BRCA* HR-associated mutations and *BRCA1*/*RAD51C* promoter methylation can predict PARPI treatment efficacy [72]. Importantly, non-*BRCA* HR-associated mutations are not consistently found in HRD-positive cases. A recent study has shown that mutations in *FANCD2*, *FANCM*, *ATM*, *PALB2*, *ATR*, or *FANCA*, which are HR-associated genes, did not strongly correlate with high scores of LOH or HRD, or platinum sensitivity. However, homozygous deletions in *CHK1* and *PTEN* were associated with high LOH scores related to HRD [73]. Another study reported that patients with mutations in HR pathway genes, such as *BRIP1*, *RAD51B*, and *CDK12*, but not *BRCA1/2*, showed similar responses to PARPIs as those harboring mutations in *BRCA1/2* [74]. It seems that different HR-associated mutations respond differently to platinum and PARPIs. Additionally, the FDA approved FoundationOne CDx for the clinical assessment of patients with mCRPC, and this test includes HR gene mutations. In the PROfound trial, PARPIs significantly improved the median OS and imaging-based median PFS of patients with mCRPC with at least one mutation in *BRCA1*, *BRCA2*, or *ATM* (detected by FoundationOne CDx) [8], [62]. Detection of HR gene mutations is technically possible, but interpreting these mutations in clinical trials remains challenging [75]. Furthermore, studies focusing on the epigenetic modifications of HR-associated genes have reported contradictory results. Some studies have reported that methylation of *BRCA1* and *RAD51C* in high-grade serous carcinoma (HGSOC) led to high HRD scores [56], [76] and correlated with good prognosis [77], [78], [79]. Epigenetic modifications of *BRCA1* were shown to display effects similar to *BRCA1/2* mutations and were involved in the genomic signatures of *BRCA* deficiency [44]. However, other studies demonstrated that *BRCA1*/*RAD51C* methylation was not an adequate biomarker for response to PARPIs [77], [80], [81], especially given that *BRCA1* or *RAD51C* hypermethylation could induce the re-expression of other proteins and partially restore HR function by demethylation [82].

Second, genomic scars reflect the state of genomic instability only at a given time and do not accurately assess the functional reconstitution of homologous recombination owing to reversion mutations and epigenetic modifications. Somatic reversion mutations of *BRCA1/2*, *RAD51C*, *RAD51D*, or *PALB2* and epigenetic modifications such as reverse promoter hypermethylation of *BRCA* or *RAD51C* result in the functional recovery of homologous recombination defects in tumors with HR-correlated mutations or HRD [83], [84], [85], [86]. In addition, these mutations lead to a weak correlation between HRD and prognosis and prediction of drug therapy efficacies. In the Triple Negative breast cancer Trial (TNT) study, the myChoice assay failed to predict platinum sensitivity in metastatic TNBC patients treated with docetaxel or carboplatin [87]. In addition, *BRCA* function restoration led to platinum resistance in *BRCA*-mutated tumors [84], [85]. Furthermore, HRD-negative patients do not necessarily have worse response to platinum/PARPI therapies. For example, in phase III of the NOVA clinical trial, niraparib improved PFS in HRD-negative patients with ovarian cancer [3]. Finally, HRD cannot detect PARPI resistance when triggered by the dysregulation of genes involved in DNA replication fork protection or other non-HR signaling pathways [86].

Third, the HRD score thresholds for different tumor types may differ. For example, the HRD scores of patients with prostate cancer were significantly lower than those of patients with ovarian cancer, and the HRD scores of patients with prostate cancer and *BRCA2* mutations were significantly higher than those of patients with *ATM* and *CHEK2* mutations. Even in the same patient, the HRD scores differed between tissues from different sites [54]. For example, a study on patients with breast cancer and brain metastases showed an evident HRD score increase in brain metastases tissues compared to their corresponding primary tumors tissues [32]. Notably, the purity of the tumor cells in the specimens can influence HRD assessment. Specifically, it was shown that a lower tumor purity made the correct assessment of HRD harder; and samples with a tumor purity > 20% could lead to a stable HRD score using the Genomic Scar Analysis (GSA) algorithm [88].

Finally, different methods of HRD assessment are non-equivalent, and there is a lack of standardized methods for the validation of the predictive efficacy and consistency of the various HRD assessment methods. An abstract from the 2020 American Society of Clinical Oncology meeting has compared several existing HRD prediction methods. Briefly, samples were defined as positive when the myChoice HRD scores exceeded the threshold (42 or 33), the percentage of LOH (%LOH) was > 16%, or mutations in HR-associated genes existed (*ATM*, *BARD1*, *BRCA1*, *BRCA2*, *BRIP1*, *CHEK2*, *MRE11A*, *NBN*, *PALB2*, *RAD51C*, and *RAD51D*). The HRD-positive results of %LOH and the 11-gene panel were compared using the myChoice HRD scores. Results showed that 19%*–*61% of HRD-positive patients detected by myChoice HRD were missed in the %LOH or 11-gene panel tests [89]. This proved that the positive results of samples obtained using different HRD detection methods are inconsistent, and even the results obtained using the same HRD detection method but different thresholds are controversial. For example, the general threshold of myChoice HRD score is 42, but some studies have demonstrated that a score of 33 was better than 42 in predicting the efficacy of PARPI therapies in EOC. Specifically, they showed that the threshold of 33 significantly correlated with improved OS after treatment compared with the threshold of 42 [90]. Also, in TNBC, the threshold score of 33 was shown to increase the sensitivity of patients to veliparib [4], [91]. However, the TBCRC 030 trial found no significant correlation between the HRD score (set at 33) and RCB/pCR after cisplatin treatment [92]. Therefore, there is an urgent need to optimize and standardize HRD assessment methods and score thresholds.

Whether the previously discussed four influences can be incorporated into assessment methods or excluded from clinical trials should be further evaluated.

## Optimization and standardization of HRD assessment

Recently, several studies have investigated the optimal definition and evaluation of HRD using phenotypes and genotypes. They discovered that the HRD status of tumors changed with time and treatment. Clinical trials results showed that the current methods for HRD detection were not consistently associated with treatment response [93]. For example, the dynamic HRD status leads to discordant responses of patients to platinum-based chemotherapies and PARPIs; consequently, HRD assessment remains an inadequate guide for planning patient therapies. Therefore, there is an urgent need to optimize existing HRD test methodologies, harmonize HRD assessment protocols, and develop optimal thresholds and time points for detecting and identifying HRD to maximize therapeutic effectiveness and minimize side effects in patients.

Currently, the status of HRD is being assessed indirectly through the detection of genomic scars, HR-associated mutations, or mutational signatures. However, there is still no unique method for identifying biomarkers that should be further included in HRD assessment. Moreover, several additional issues related to the optimization and harmonization of HRD assessment need to be addressed. First, we needed to identify the optimal sequencing methods for HRD assessment. High-density SNP arrays of the whole genome and genomic SNP backbone probes using NGS, genome-wide WGS, and whole-exome sequencing (WES) can be used to detect and calculate genomic scars and compute genomic instability scores. In addition, WGS data can be used to analyze genomic features such as microhomologous deletions, Catalogue of Somatic Mutations in Cancer (COSMIC) signatures, and structural variants [29].

WGS data can more accurately determine mutational signatures such as large-scale and structural rearrangements [94]. However, the clinical application of WGS assays is limited by their high upload DNA volume, high sequencing data volume, and high costs. Hence, the software tool ShallowHRD was developed to partially address these shortcomings. ShallowHRD is based on large-scale genomic alterations detected using low-coverage WGS with 1× reading depth, providing HRD detection with 90.5% specificity and 87.5% sensitivity. In addition, the HRD scores of shallowHRD showed good correlations with those obtained using WGS [95], [96]. Furthermore, low-coverage WGS can detect copy number alterations in cell-free DNA (blood), which correlates well with copy number alterations in tumor samples [97]. Therefore, shallowHRD is a cost-effective and promising method for predicting the efficacy of platinum-based drugs and PARPI treatments.

The WES data can also be used to detect HRD-induced mutational signatures. However, the HRD-induced mutations per sample detected by WES are 100 times less than those detected by WGS. In addition, the number of detected deletions by WES are near or below the threshold for HRD detection. In particular, microhomology-mediated deletions, which are strongly associated with HRD-induced mutational signatures, cannot be accurately assessed using WES data [29]. WES cannot detect non-coding regions and structural variants; hence, many driver events of cancer occurring in non-coding regions may not be detected [86]. However, the HRDetect values between WES and WGS display an overall good correlation (*r* = 0.71) [98]. Therefore, given the sample volume, data volume, and somatic mutations that need to be detected, a high-depth WES plus high-density SNPs method can be used to comprehensively detect germline/somatic HR mutations and assess the genomic instability status of tumors. As the cost of WES testing decreases and the testing technology continues to mature, WES testing has the potential to become an accurate assessment method for HRD. This may also guide the planning of platinum-based chemotherapies and PARPI therapies.

In addition to the detection methods mentioned above, Signature Multivariate Analysis (SigMA) used a likelihood-based measure and machine learning techniques to assess the mutational signature Sig3 induced by defects in HR based on targeted gene panels. Patients with ovarian cancer and HRD defined by SigMA showed a significantly longer OS after platinum therapy. Sig3-positive patients without *BRCA1/2* mutations had a similar OS to patients with *BRCA* mutations; moreover, the hazard ratio of Sig3-positive *versus* Sig3-negative patients was found to be 0.53 [95% confidence interval (CI) = 0.37–0.74; *P* < 0.001]. SigMA applications are promising and may enhance the benefit of patients from platinum-based and PARPI treatments, because targeted gene panels are the most prevalent genetic testing platforms used in clinical practice [99].

The second matter requiring further optimization is the samples that are most suitable for detecting HRD. Blood is generally used to detect germline mutations, whereas other tissues are used to detect somatic mutations [100]. FFPE sections, which are easy to store and transport, have been generally used to detect SNP–HRD, WES–HRD, and WGS–HRD. Tumor tissues are difficult to sample at multiple time points to continuously monitor HRD changes, whereas blood easily allows this practice. However, there are currently relatively few clinical trials using blood samples for HRD detection, except those focused on germline mutations.

Finally, we need special validation systems for HRD assessment in Chinese patients. The HRD threshold should be verified based on the efficacy of platinum-based and PARPI-added therapies in patients. Two HRD assays, myChoice CDx and FoundationFocus CDx*_BRCA_*_LOH_, are primarily based on genetic data obtained from Caucasian populations. An observational study based on real-world evidence showed that PARPIs could significantly improve PFS among HRD-positive Chinese patients with ovarian cancer, and it proved that HRD can independently predict the efficacy of PARPI treatments in Chinese patients with ovarian cancer [101]. However, additional evidence from clinical trials is required to determine whether assessment of HRD is suitable in Chinese patients. Currently, China has not yet approved clinical HRD tests based on genomic scars or HR-associated mutational signatures. Therefore, there is a need to develop HRD tests that can be used in the Chinese clinical practice. Hence, HRD tests should be designed in accordance with the genetic profiles of the Chinese population. Furthermore, the HRD score thresholds of the Chinese population should be optimized based on the association between the genomic damage status of the Chinese population and mutations in HR-associated genes such as *BRCA*. In addition, thresholds should also be determined based on efficacy of response to treatment.

**Figure 3:**
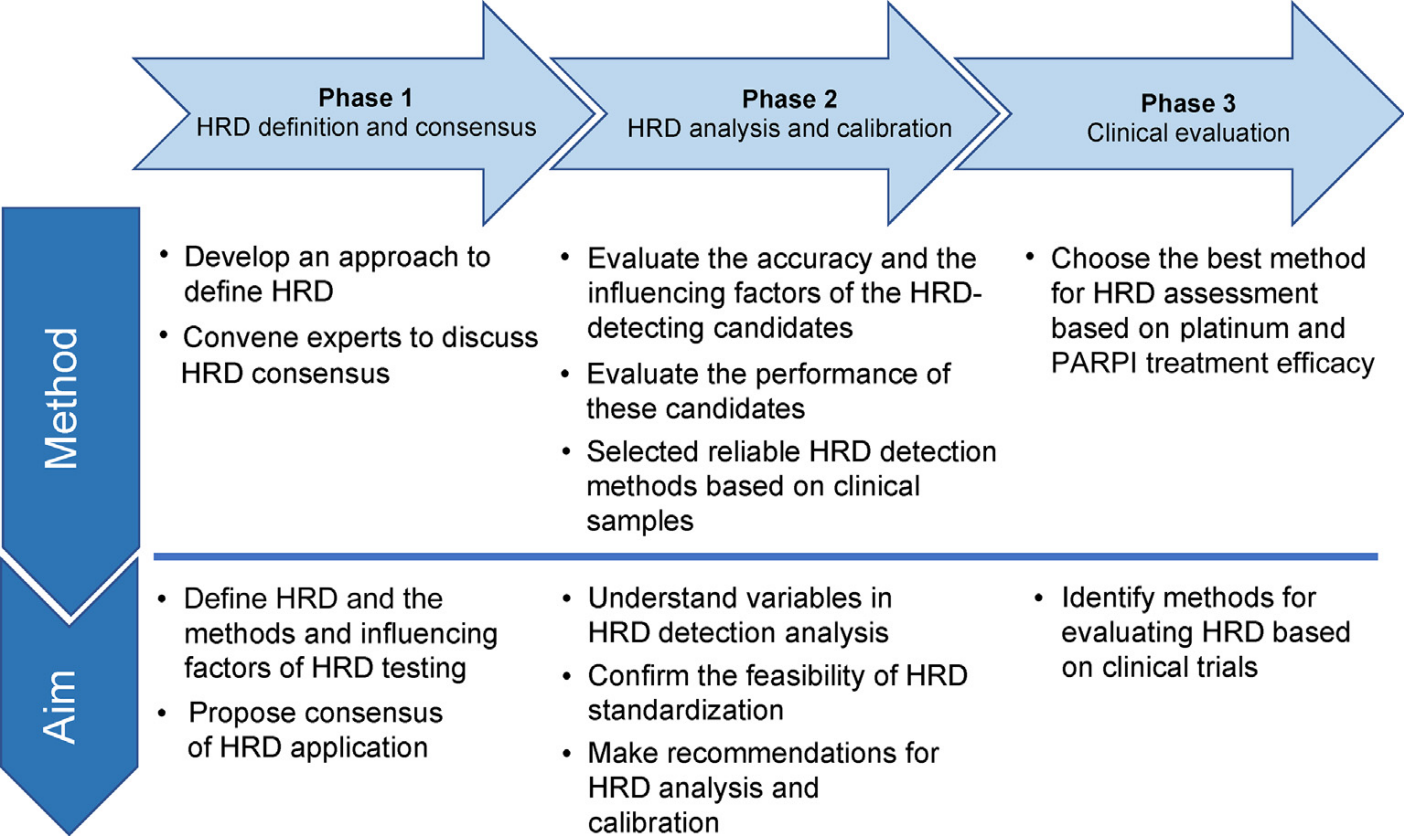
**The flow chart of the Chinese HRD Harmonization Project** The Chinese HRD Harmonization Project, which will be jointly implemented by the National Cancer Center / Cancer Hospital, Chinese Academy of Medical Sciences, the Pathology Committee of the Chinese Anti-Cancer Association, and the China National Institutes for Food and Drug Control, aims to standardize the definition, testing methods, and reports of HRD and promote the development and application of HRD as a biomarker in cancer clinical trials. The project was divided into three phases, as shown in the figure. PARPI, poly(ADP-ribose) polymerase inhibitor.

Based on the above principles, the Chinese HRD Harmonization Project, which will be jointly implemented by the National Cancer Center / Cancer Hospital, Chinese Academy of Medical Sciences, the Pathology Committee of the Chinese Anti-Cancer Association, and the China National Institutes for Food and Drug Control, aims to standardize the definition, testing methods, and reports of HRD and promote the development and application of HRD as a biomarker in clinical trials. This project is divided into three phases (Figure 3). Phase 1: HRD definition and consensus. This phase aims to define HRD and the methods and influencing factors of HRD detection and to propose a consensus on HRD applications. The plan is to develop a comprehensive analytical approach that aims to define the use of HRD and HR calls; propose a common language around the use of HRD; and convene clinical, biomedical, and corporate experts to debate the HRD consensus. Phase 2: HRD analysis and calibration. This phase aims to understand the variables in the HRD detection analysis, confirm the feasibility of HRD standardization, and make recommendations for HRD analysis and calibration. Specifically, standards are used to evaluate the accuracy and influencing factors of HRD detection candidates. Additionally, standard datasets will be used to evaluate the performance of these candidates and understand the influence of certain variables, such as the number of SNPs and genomic distribution in the results. HRD detection methods with relatively reliable performance among all candidates (*e.g.*, SNP panel, WES, and WGS) will then be evaluated using clinical samples. Phase 3: Clinical evaluation of the HRD tests. This last phase aims to identify methods for evaluating HRD based on clinical trials. In this phase, we will assess the predictive value of HRD status using different weights and correction methods based on platinum and PARPI treatment efficacy. Our final aim is to identify the best method for assessing HRD.

## Conclusion

The standardization of HRD detection and its clinical applications still have a long way to go. However, the relevance and wide range of HRD applications, as well as the availability of new technologies and methods to improve HRD standardization and optimization, make this an exciting journey. For example, single-cell genomics can resolve intratumoral heterogeneity to further optimize the factors included in HRD detection [102]. The future of this field will be defined by the rapid development of genetic testing technologies, continuous improvement of HRD assessment methods, and multidisciplinary involvement of clinicians, pathologists, molecular testers, clinical pharmacists, and tumor biology experts in tumor precision medicine. Additionally, comprehensive HRD detection methods that take into consideration HR gene mutations, mutational signatures, and reversion mutations will further be developed to monitor changes during cancer development and accurately predict the efficacy of treatments. We envision that precise assessment of HRD will further improve tumor diagnosis and treatment to benefit an increasing number of tumor patients.

## Competing interests

Lin Gao, Xin Yi, and Shuangfeng Shi are current employees of Geneplus. All the other authors have declared no competing interests.

## CRediT authorship contribution statement

**Wenbin Li:** Conceptualization, Investigation, Writing – original draft, Writing – review & editing, Visualization, Funding acquisition. **Lin Gao:** Investigation, Writing – original draft, Writing – review & editing, Visualization. **Xin Yi:** Project administration. **Shuangfeng Shi:** Project administration. **Jie Huang:** Project administration. **Leming Shi:** Project administration. **Xiaoyan Zhou:** Project administration. **Lingying Wu:** Project administration. **Jianming Ying:** Conceptualization, Funding acquisition. All authors have read and approved the final manuscript.
